# Nurse led titration of guideline-directed medical therapy for heart failure: an operative flow chart

**DOI:** 10.1093/eschf/xvag223

**Published:** 2026-10-06

**Authors:** Mauro Gori, Michela Barisone, Renata De Maria, Luca Fazzini, Marco Spagnolin, Vittoria Rizzello, Matteo Marchesi, Daniela Pini, Donatella Radini, Emilia D’Elia, Norma Caples, Pietro Ameri, Samuele Baldasseroni, Andrea Di Lenarda, Marco Marini, Annamaria Municinò, Alessandro Verde, Michele Senni, Samuela Carigi

**Affiliations:** Cardiology Unit, Cardiovascular Department, ASST Papa Giovanni XXIII, Piazza OMS 1, 24128 Bergamo, Italy; University of Milano-Bicocca, Piazza dell'Ateneo Nuovo 1, 20126 Milano MI, Italy; Clinical Epidemiology and Research Center, Humanitas Research Hospital, Pieve Emanuele, MI, Italy; Dipartimento di Cure Primarie, ASST Fatebenefratelli Sacco, Milan, Italy; Cardiology Unit, Cardiovascular Department, ASST Papa Giovanni XXIII, Piazza OMS 1, 24128 Bergamo, Italy; Cardiology Unit, Cardiovascular Department, ASST Papa Giovanni XXIII, Piazza OMS 1, 24128 Bergamo, Italy; U.O.C. Cardiologia, A.O. San Giovanni Addolorata, Roma, Italy; Chief of Legal Medicine Unit—ASST Papa Giovanni XXIII, Bergamo, Italy; S.C. Cardiologia, Fondazione IRCCS San Gerardo dei Tintori, Monza, Italy; SC Patologie Cardiovascolari, Dipartimento Specialistico Territoriale, Azienda Sanitaria Universitaria Giuliano-Isontina (ASUGI), Trieste, Italy; Cardiology Unit, Cardiovascular Department, ASST Papa Giovanni XXIII, Piazza OMS 1, 24128 Bergamo, Italy; University of Milano-Bicocca, Piazza dell'Ateneo Nuovo 1, 20126 Milano MI, Italy; Advanced Nurse Practitioner, Heart Failure Service, Department of Cardiology, University Hospital Waterford, Ireland; U.O.C Clinica di Malattie dell’Apparato Cardiovascolare e UTIC, IRCCS Ospedale Policlinico San Martino, Genova, Italy; Dipartimento di Medicina Interna e Specialità Mediche, Università di Genova, Genova, Italy; Cardiologia e Medicina Geriatrica, Azienda Ospedaliero-Universitaria Careggi, Firenze, Italy; S.C. Patologie Cardiovascolari, Dipartimento Specialistico Territoriale, Azienda Sanitaria Universitaria Giuliano-Isontina (ASUGI), Trieste, Italy; S.O.D. Cardiologia-UTIC, A.O.U. delle Marche, Ancona, Italy; S.C. Cardiologia, Ospedale San Bartolomeo, ASL 5, Sarzana, SP, Italy; S.C. Cardiologia 2—Insufficienza Cardiaca e Trapianti, Dipartimento Cardiotoracovascolare ‘A. De Gasperis’, ASST Grande Ospedale Metropolitano Niguarda, Milan, Italy; Cardiology Unit, Cardiovascular Department, ASST Papa Giovanni XXIII, Piazza OMS 1, 24128 Bergamo, Italy; University of Milano-Bicocca, Piazza dell'Ateneo Nuovo 1, 20126 Milano MI, Italy; U.O.C Cardiologia, Ospedale Infermi, AUSL della Romagna, Rimini, Italy

**Keywords:** Heart failure, Pharmacological up-titration, Guideline-directed medical therapy, Heart failure specialist nurse

## Abstract

**Background and Aims:**

Titration of guideline-directed medical therapy (GDMT) in heart failure (HF) is recommended by current guidelines. Recent data show that titration is not performed in most patients because of barriers related to healthcare systems, patient factors, and therapeutic inertia, leading to worse patient outcomes.

**Methods and Results:**

Nurse-led titration of GDMT for HF may increase the uptake of recommended HF therapies and doses. In Italy, only a few seminal experiences regarding multi-professional management of HF therapy have been reported, and huge disparities regarding this issue persist across European countries. This article describes the operational aspects required to implement a nurse-led GDMT titration program. Italian medical–legal aspects are also discussed, and an operational flow chart is provided. The main prerequisites are dedicated training and the adoption of management protocols, which, pending national educational plans and protocols, should start at the level of local institutions. These requirements should be complemented by detailed medical prescriptions specifying, for each patient, the timing and methods of titration, any other assessments to be performed, and the resulting actions.

**Conclusions:**

A multi-professional strategy to manage HF patients involving nurse-led titration of GDMT for HF is suggested as a possible way to improve guideline uptake in real-world practice. This paper aims to describe how to implement it in daily practice in countries such as Italy, where nurse-led titration is not yet standard of care.

## Graphical Abstract

**Graphical Abstract:**
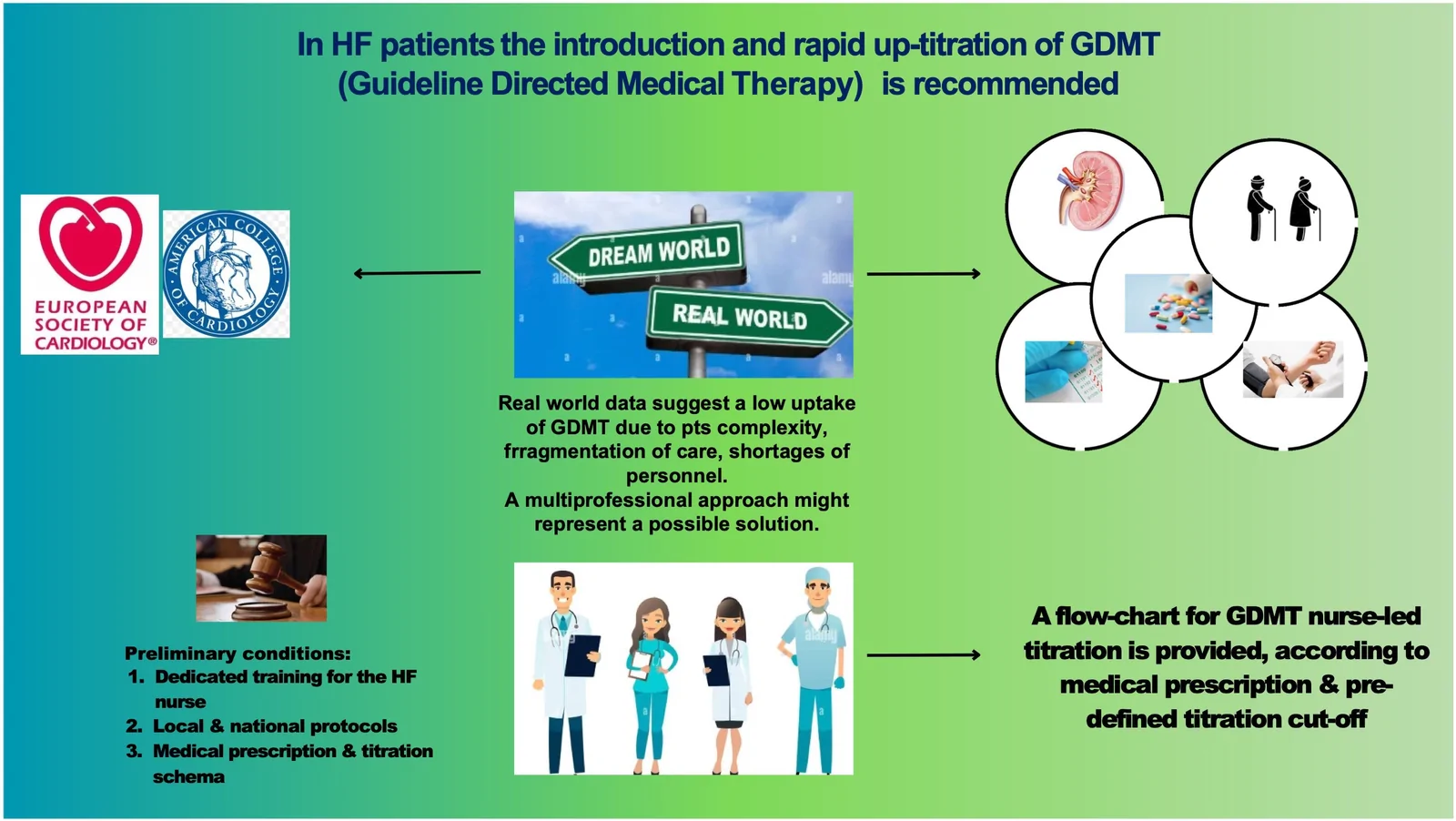

Highlights box

- The role of specialist nurses in heart failure (HF) is not yet formally recognized and implemented in several European countries, including Italy. Various studies show that specialist HF nurses promote self-management and reduce hospitalizations, improving the efficiency of health care.
- It is hoped that the titration of Guideline-directed medical therapy (GDMT) for HF by specialist HF nurses will be implemented in outpatient HF clinics across Europe, to pursue a multidisciplinary care approach, in collaboration with the cardiologist.
- The skills of specialist HF nurses must be supported by dedicated training, as well as on-the-job experience.
- This article provides an operative flow chart regarding nurse-led titration of GDMT in Italy and might help to spread this management strategy to those European countries where multi-professional management is not yet standard of care.

## Introduction

The rapid initiation and titration of guideline-directed medical therapy (GDMT) in patients with heart failure (HF) are recommended by current guidelines.^1–7^ GDMT refers to the evidence-based pharmacological treatment recommended by international guidelines for each HF phenotype. In heart failure with reduced ejection fraction (HFrEF), GDMT comprises four complementary disease-modifying classes—an angiotensin receptor-neprilysin inhibitor (ARNI), or an angiotensin-converting enzyme inhibitor/angiotensin receptor blocker when ARNI is not feasible; an evidence-based β-blocker; a mineralocorticoid receptor antagonist (MRA); and a sodium-glucose cotransporter 2 inhibitor (SGLT2i). These therapies should be initiated rapidly and subsequently titrated to target or maximally tolerated doses. Their complementary mechanisms provide additive and early reductions in mortality and HF hospitalization, making broad implementation of all four pillars preferable to the sequential maximization of a single class.

However, due to barriers related to healthcare systems, therapeutic inertia, and patient factors, currently only a minority of patients is treated with GDMT appropriately, resulting in worse clinical outcomes.^8,9^ Patient-level factors include low blood pressure, renal dysfunction, hyperkalaemia, bradycardia, residual congestion, frailty, multi-morbidity, polypharmacy, and real or perceived intolerance; medication adherence, patient preferences, and socioeconomic factors may also influence implementation.

In particular, dose titration remains largely inadequate.^10–14^ Frequent reassessment of symptoms, vital signs, renal function, and electrolytes is required during titration, but limited follow-up capacity and therapeutic inertia often delay or prevent dose escalation. In EVOLUTION HF, only 28.2% of patients initiating sacubitril/valsartan, 7.2% initiating β-blockers, and 5.1% initiating MRA achieved target doses within 12 months.^8^

Contemporary Italian registries confirm that important gaps persist, although rates vary according to setting. In OpTIMa-HF, 46.9% of HFrEF outpatients received all four pillars and only 1.1% of patients on quadruple therapy achieved the target doses.^14^ In the BRING-UP-3 ambulatory cohort, 65% received all four pillars.^13^ In OPTIPHARM-HF, the proportion of patients receiving quadruple therapy increased from 47% at baseline to 64% after the first specialist assessment, while fewer than 60% achieved at least 50% of the target doses.^15^ Italian data are broadly consistent with those reported in other European countries: quadruple therapy was prescribed to 60% of patients in Sweden and to 66% in the Netherlands, although only 1.3% of patients achieved the target dose of all four drug classes.^12^

## Rationale for nurse-led GDMT titration

One potential solution is multi-professional HF management with the active involvement of nursing staff in the therapeutic titration process. Nurse-led titration has been shown to improve outcomes in patients with HF (*Graphical abstract*).^16,17^

In the UK and Scandinavia, advanced nursing professionals (Advanced Nurse Practitioners and Independent Nurse Prescribers) have considerable autonomy in managing drug therapies.^18,19^ This high degree of autonomy is based on three fundamental prerequisites: specific training on the drug therapies to be managed, therapy management protocols approved by national and/or local institutions, and detailed medical prescriptions for individual case management. In 2016, the European Society of Cardiology published the HF nurse curriculum to standardize nurses’ knowledge, competences, and behaviour, underscoring that a multidisciplinary approach is key to success.^20^ However, the role of HF nurses is not always standardized and recognized across Europe.^21^ To contribute to discussions on how best to promote active nurse involvement in GDMT optimization, we describe the Italian experience.

In Italy, despite the absence of specific regulations on drug titration, current legislation does not preclude nurses from performing this activity, provided that they adhere to principles of good practice, such as dedicated training and the adoption of management protocols. Pending national courses and protocols, these requirements, together with detailed medical prescriptions specifying the timing and methods of titration on a case-by-case basis, should initially be implemented at the local institutional level. The purpose of this paper is to provide an operational flow chart regarding nurse-led titration of GDMT, applicable in Italy and throughout other European countries.

The original contribution of this paper is to combine clinical monitoring, a 12-week workflow, shared nurse-cardiologist decision-making, and medico-legal governance in a single operational model tailored to Italy but designed for adaptation to other European healthcare systems.

## How to titrate GDMT: tips and tricks

In patients with HFrEF, drug titration involves integrating the four disease-modifying therapies and diuretics to maximize prognostic and symptomatic benefit for the patient.^10–14^ In the absence of side effects and/or patient intolerance, HF drugs should be uptitrated to the target doses, which were demonstrated to significantly impact outcomes in pivotal studies.^7^ Guidelines recommend the rapid and close initiation of the four pillars, but the sequence and timing of titration are left to clinical discretion, which adds another element of complexity.^1–5^ If titration to target doses is not possible due to patient intolerance, which is far from uncommon, quadruple therapy with reduced RASIs and β-blockers should be preferred to omitting one class in order to maximize another.^22^ Real-world evidence has recently documented benefits in cardiac remodelling, quality of life, and natriuretic peptide levels, even when target ARNI doses are not achieved.^23^ In HFrEF patients with worsening HF, vericiguat may also be safely considered for its proven efficacy.^5^

*Table 1* depicts the clinical parameters to be integrated for a safe drug titration, as indicated in the trials. Caution should be exercised in particular in patients older than 75 years of age, in whom comorbidities and frailty augment management complexity.^17–24^

**Table 1:** Parameters to be monitored during GDMT titration

Drug class	Clinical parameters to initiate and titrate	Adverse events	Management/Interventions	Notes
ACEi/ARB/ARNI	SBP >100 mmHg eGFR >30 mL/min/1,73 mq K+ <5.5 mmol/L	Asymptomatic hypotension Symptomatic hypotension (e.g. dizziness, confusion) Severe hypotension/cardiogenic shock; eGFR decrease Serum potassium >5.5 mmol/L Serum potassium >6 mmol/L Cough Angioedema	Continue therapy. 1. Assess volume status and reduce/stop diuretic if no congestion. 2. Review other BP-lowering drugs (e.g. calcium antagonists, nitrates, diuretics). 3. If still symptomatic: a) Temporarily reduce ACEi/ARB/ARNI or beta-blocker dose; b) Review patient shortly and continue reduction if symptoms persist. Refer to ER immediately. Assess volume status, review nephrotoxic drugs, reduce or stop dose if needed. Review K+ supplements, consider potassium binders, reduce or stop ACEi/ARB/ARNI if needed Consider potassium binders, stop ACEi/ARB/ARNI, Consider switch from ACEi to ARB. Angioedema → discontinue and refer specialist.	Diuretic dose can be reduced anytime if euvolaemic (unless previously worsened symptoms). Monitor BP, renal function and potassium closely (2 weeks) after dose titration.
Beta-blockers	HR >60 bpm SBP >100 mmHg NT-proBNP levels not indicative of worsening congestion (↑ < 10%)	Asymptomatic hypotension; Symptomatic hypotension; Severe/shock hypotension; Asymptomatic bradycardia (50–60 bpm); Symptomatic bradycardia (<50 bpm); Fatigue; Cold extremities Fluid overload: ↑ by ≥10% of NT-proBNP (worsening congestion)	Continue therapy. Assess volume status, adjust diuretic dose, review other anti-hypertensives, reduce beta-blocker if needed. Emergency evaluation Continue therapy. 1. Perform ECG; 2. Review other HR-lowering drugs (e.g. digoxin, amiodarone); 3. If persists, reduce dose. Consider switch to vasodilating beta-blocker. Do not titrate, consider ↑ diuretic dose	Avoid abrupt withdrawal. Monitor HR and BP regularly. Diuretic dose can be reduced anytime if euvolaemic.
MRA	SBP >100 mmHg eGFR >30 ml/min/1,73 mq K+ <5.5 mmol/L	Asymptomatic hypotension; Symptomatic hypotension Serum potassium >5.5 mmol/L Serum potassium >6 mmol/L eGFR decrease Gynecomastia (spironolactone) Dehydration Congestion (fluid overload)	Continue therapy. Assess volume status, review other anti-hypertensives, reduce dose if needed. Monitor serum potassium and eGFR, reduce dose or stop MRA if needed, consider potassium binders. Stop MRA and consider potassium binders. eGFR decrease ≤30% → continue therapy, monitor. eGFR decrease >30% → assess volume status, consider reduction dose or discontinuing Switch to eplerenone. Reduce diuretic or MRA dose, ensure hydration. Monitor symptoms and renal function, adjust diuretics or temporarily stop if recently initiated.	Avoid NSAIDs. Monitor renal function and electrolytes regularly.
Diuretics		Hypotension; Dehydration; Hyponatremia; Hypokalaemia Congestion (fluid overload) Gout/hyperuricemia	Reduce dose, check volume status. Reduce or withhold dose until euvolaemic. Correct electrolytes and adjust therapy. Increase diuretic dose 50–100% to achieve 0.5–1 kg/day weight loss; Monitor uric acid, consider therapy adjustment.	Monitor weight changes (≥2 kg in 2 days). Reassess after correction of volume or electrolytes.
SGLT2i	SBP >100 mmHg eGFR >25 mL/min/1,73 mq	Symptomatic hypotension Increase in serum creatinine >30% within 4 weeks of initiating, development of ketones or elevated lactate Genito-urinary infection	Assess volume status, review other anti-hypertensives, reduce dose of diuretic Consider temporary suspension Reinforce genital hygiene. Consider treating genital micotic infection with a single dose of fluconazole 150 mg	Early rise in serum creatinine is an anticipated effect of the drug; Counsel patient to temporarily hold SGLT2i if acutely unwell, and stop before surgery or procedures.
Vericiguat	SBP ≥100 mmHg eGFR ≥15 mL/min/1,73 mq	Symptomatic hypotension	Assess volume status, review other anti-hypertensives, reduce dose if needed.	

ACEi, angiotensin converting enzyme inhibitors; ARB, angiotensin receptor blockers; ARNI, angiotensin and neprilisyn receptor inhibitors; SBP, systolic blood pressure; eGFR, estimated glomerular filtration rate; HR, heart rate; ER, emergency room; MRA:

Congestion should be accurately assessed by integrating symptoms and signs and/or biomarkers suggesting fluid accumulation.^25,26^ The STRONG-HF study used a 10% increase in NT-proBNP from the euvolemic ‘dry’ value for each patient as an indicator of worsening congestion precluding β-blocker titration.^7^

Conversely, pharmacological management in patients with HFmrEF and HFpEF involves less dose titration, since SGLT2 inhibitors, which have a class I indication in guidelines, do not require up-titration. These agents should be prescribed to all HFmrEF and HFpEF patients without contraindications and/or intolerance.^3^ Modulation of diuretic therapy based on the degree of congestion remains important in this population while finerenone, as recently documented in the FINEARTS-HF trial, may provide additional benefit on mortality from cardiovascular cause and hospitalization for HF.^27^

Although no randomized trial evidence supports RASI and β-blocker in patients with HFmrEF and HFpEF, data from the STRONG-HF and the Italian Network in HF registry suggest a beneficial effect of both drug classes in this population.^28,29^ However, since RASI and β-blocker are indicated to manage risk factors and comorbidities in HFmrEF and HFpEF patients, the dose to be achieved will depend on blood pressure or heart rate control, with no definite titration needs.

## Medical–legal aspects

From a practical standpoint, the active involvement of an HF nurse in implementing a drug titration programme, within their respective scopes of practice, can be achieved in Italy through two main approaches. The first requires a stringent pre-emptive medical prescription based on parameters emerging from the clinical history and physical examination, that details the drug and dose, method, and rate of administration, and titration, to which the nurse must adhere. Should any new clinical event or physical finding differ from those detailed in the initial prescription, the nurse must seek medical intervention. The second approach involves a consultation at the time of titration, even remotely, between the nurse and the cardiologist, resulting in a joint report.

The clinical core of the pathway is potentially transferable across healthcare systems. However, each centre must adapt the model to national and local regulations governing nursing scope of practice, independent or supplementary prescribing, professional liability, access to laboratory testing, electronic documentation, and remote consultation. Where nurses cannot prescribe independently, titration can be implemented through a detailed prospective medical prescription or real-time cardiologist approval.

## Pivotal Italian experiences

Nowadays, in Italy, there are few preliminary experiences regarding nurse-led GDMT titration.

At the Romagna Local Health Authority (AUSL), an intensive multi-professional medical and nursing management programme for patients with HF has recently been implemented. Patients are assessed by the nurse at 2, 6, and 9 weeks, and by the physician (sometimes via telemedicine) at 4 and 12 weeks. The nurse takes charge of the patient at discharge and during the first outpatient visit in a dedicated nursing clinic and checks encoded clinical and instrumental parameters. They may then recommend GDMT titration or even initiating any missing drug classes. Any therapeutic changes are validated by the physician, who signs the new prescription.

At the Fondazione IRCCS San Gerardo in Monza, a nurse-led titration programme for patients admitted with acute HF has recently started. The follow-up consists of four visits (1, 2, 3, and 6 weeks after discharge), the first and last of which are conducted in person and followed by a cardiologist visit, whereas the others are conducted via telemedicine. The programme starts upon discharge with a detailed medical prescription for the titration schedule of ARNI/RASI and beta-blockers. A preliminary data analysis showed that a high proportion of patients achieved GDMT target doses 6 weeks after discharge.

At the Cardiovascular Centre, University Hospital and Health Services of Trieste, an early post-discharge community-based HF management programme has been in place for years. This programme involves telephone contacts and interventions, a structured interview recorded in the electronic medical record, and subsequent scheduling of outpatient medical or nursing care within 30 days of discharge. The timing is personalized based on the patient's degree of severity/instability. The nursing staff, appropriately trained in the field and in accordance with shared algorithms, can propose the introduction of new medications and dose adjustments. The care plan is shared with the referring cardiologist, and the report is jointly signed.^30^

## Operative flow chart and transferability of the model

*Figure 1* provides an operational flowchart for a 12-week joint medical-nursing follow-up.

**Figure 1:**
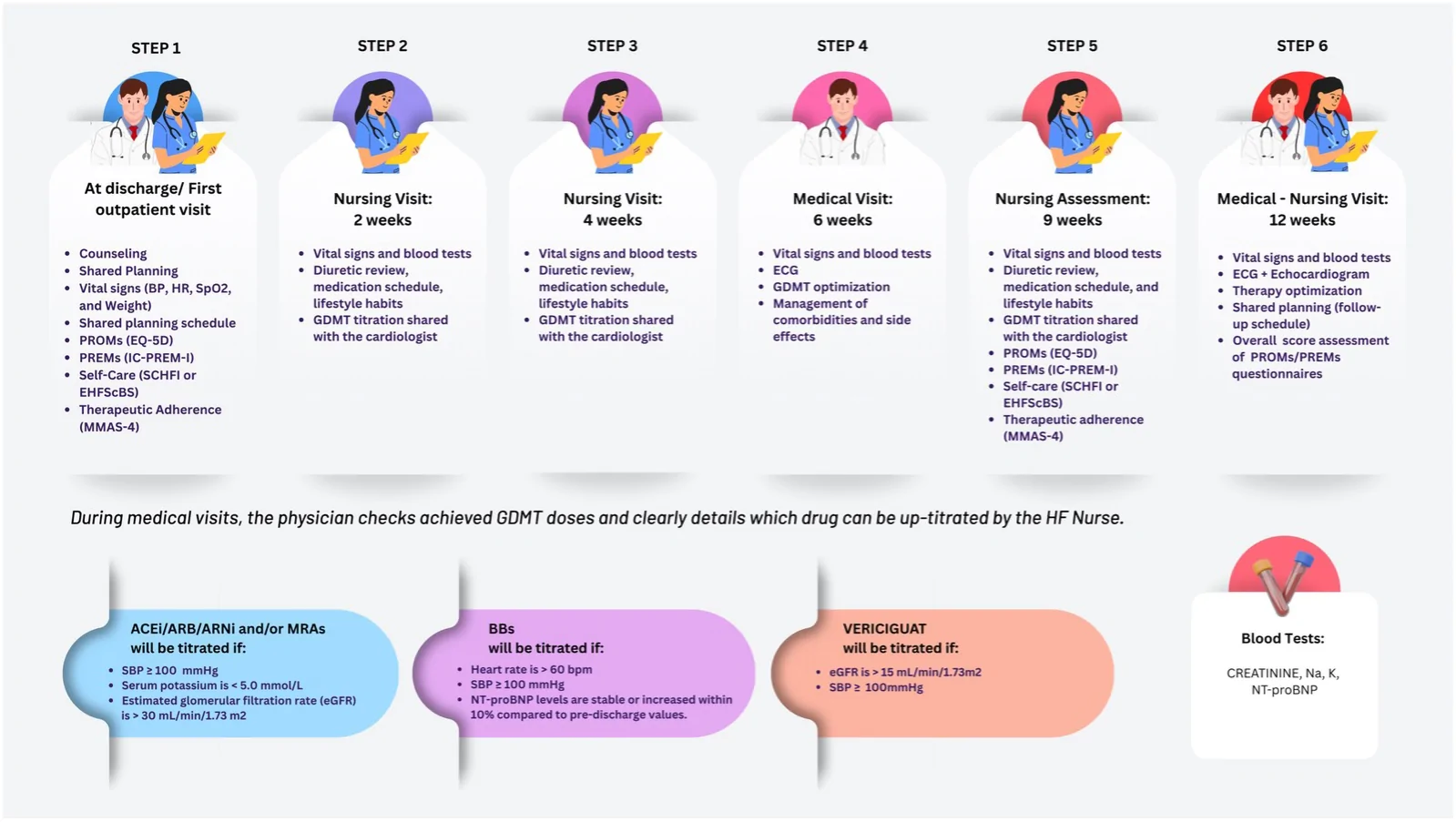
Operational flow chart for the implementation of an integrated approach to GDMT titration in clinical practice

The patient enters the pathway at discharge after hospitalization for acute HF. During nursing visits at 2 and 4 weeks, clinical and laboratory parameters (electrolytes, renal function, natriuretic peptides) are assessed. Based on these findings, after approval from the referring cardiologist, the nurse titrates GDMT, while the nurse and cardiologist jointly sign the visit report. At 6 weeks, the patient is re-evaluated by the cardiologist, who prescribes any additional therapeutic adjustments as necessary. This is followed by a further nursing assessment at 9 weeks. At 12 weeks, a joint medical-nursing clinical and instrumental reassessment verifies the disease trajectory and the need for any device. Subsequent follow-up is personalized based on the individual patient's risk stratification.^17^

The flow chart is a proposed organizational model rather than the report of a single prospective implementation cohort; consequently, it is not associated with a predefined number of patients or a validated nurse-to-patient ratio. As a pragmatic minimum, two specifically trained HF nurses should be involved. This recommendation is an organizational principle rather than an evidence-based staffing standard.

Dissemination should proceed through institutional endorsement, standardized education based on the HFA nurse curriculum, local adaptation of the protocol, pilot implementation, and prospective audit. Regional and national HF networks can facilitate shared training, benchmarking, and iterative refinement.

## Conclusions

The benefits of appropriate medical therapy for HF, both in terms of drug classes and doses, have been widely demonstrated. However, real-world practice is misaligned with this evidence; in particular, drug doses remain largely suboptimal.

In the setting of drug titration, some European experiences have demonstrated that the involvement of dedicated and trained HF nursing staff can optimize patient care, leading to improved outcomes. Although Italian legislation does not preclude nurse-led drug titration, considerable mistrust and fear among healthcare professionals still surround nursing staff involvement in this activity. The present paper, by detailing an operational methodology, aims to outline how to develop a multidisciplinary management pathway for HF in countries such as Italy, where nurse-led titration is not yet standard of care.

## Declarations

### Data Availability

No data were generated or analysed for this manuscript.

### Pre-registered Clinical Trial Number

None supplied.
